# Evaluation of the association of *C5* with neovascular age-related macular degeneration and polypoidal choroidal vasculopathy

**DOI:** 10.1186/s40662-019-0161-2

**Published:** 2019-11-07

**Authors:** Ke Liu, Li Ma, Timothy Y. Y. Lai, Marten E. Brelen, Pancy O. S. Tam, Clement C. Tham, Chi Pui Pang, Li Jia Chen

**Affiliations:** 1 Department of Ophthalmology and Visual Sciences, The Chinese University of Hong Kong, Hong Kong, China; 2 Department of Ophthalmology and Visual Sciences, Prince of Wales Hospital, Hong Kong, China

**Keywords:** Age-related macular degeneration, Polypoidal choroidal vasculopathy, Complete component 5, C5, Genetic association, Single-nucleotide polymorphism

## Abstract

**Background:**

Neovascular age-related macular degeneration (AMD) and polypoidal choroidal vasculopathy (PCV) are sight-threatening maculopathies with both environmental and genetic risk factors. We have previously shown relative risks posed by genes of the complement pathways to neovascular AMD and PCV.

**Methods:**

In this study, we investigated the haplotype-tagging single nucleotide polymorphisms (SNPs) in the *complement component 5* (*C5*) gene in 708 unrelated Chinese individuals: 200 neovascular AMD patients, 233 PCV patients and 275 controls. Six tagging SNPs in *C5* were genotyped. Univariate single SNP association analysis, haplotype-based association analysis and gene-gene interaction analysis between *C5* and other AMD-associated genes were performed.

**Results:**

The results revealed none of the six tagging SNPs of the *C5* gene had a significant association with neovascular AMD or PCV (*P* > 0.05). We also found insignificant haplotype-based association, and no significant SNP-SNP interaction between *C5* and other genes (including *C2*-*CFB*-*RDBP*-*SKIV2L*, *SERPING1*, *CETP*, *ABCG1*, *PGF*, *ANGPT2*, *CFH* and *HTRA1*) for neovascular AMD and PCV.

**Conclusions:**

This study showed no statistical significance in the genetic association of *C5* with neovascular AMD or PCV in a Hong Kong Chinese population. Further studies in large samples from different populations are warranted to elucidate the role of *C5* in the genetic susceptibility of AMD and PCV.

## Background

Neovascular age-related macular degeneration (AMD), characterized by choroidal neovascularization (CNV) at the macular region, is a leading cause of irreversible blindness among the elderly in developed countries [1]. The proportion of neovascular AMD in advanced AMD is higher in Asians than in Caucasians [2]. In the Chinese population it is the major AMD subtype that leads to central vision loss [3]. Polypoidal choroidal vasculopathy (PCV), with characteristic inner choroidal vascular networks ending in polypoidal lesions, is also a sight-threatening maculopathy. PCV belongs to a spectrum of conditions known as pachychoroid, characterized by choroidal thickening that includes central serous chorioretinopathy and PCV [4, 5]. PCV has been considered as a subtype of neovascular AMD since PCV has overlapping clinical features with neovascular AMD, such as retinal pigmented epithelium (RPE) detachment, submacular hemorrhage, fluid and exudates [6, 7]. However, significant differences between neovascular AMD and PCV have also been observed in epidemiology, clinical course and response to treatment [1, 8–10]. The incidence of PCV in the overall neovascular AMD patients was reportedly about 24.5 to 54.7% in Asians [1, 11–13], comparing to approximately 8.7% in Caucasians [13, 14]. In treatment, neovascular AMD responds well to anti-vascular endothelial growth factor (anti-VEGF) monotherapy, whilst PCV usually requires combined anti-VEGF and photodynamic therapy [13]. The plasma inflammaging profiles are also different between patients with PCV and neovascular AMD [15]. Therefore, whether PCV is a subtype of AMD or a different disease category remains an open question that needs more profound review and investigation.

Both neovascular AMD and PCV are multifactorial in etiology, resulting from the interactions of aging, genetic and environmental factors. In the past decade, molecular genetic studies, including candidate gene analysis, genome-wide association studies (GWAS), and exome-wide association studies (EWAS), have identified single-nucleotide polymorphisms (SNPs) in over 30 genes that are associated with AMD [16–22]. Among them, the *complement factor H (CFH)* gene and the *ARMS2/HTRA1* locus were the most strongly associated with AMD [16, 17, 23, 24] and PCV [25] in different populations, although SNPs at the *ARMS2/HTRA1* locus had stronger effect sizes in neovascular AMD than in PCV [25]. In contrast, an exome-wide association study identified a rare variant (p.Lys329Arg) in the *FGD6* gene to be significantly associated with PCV but not with neovascular AMD [26]. These findings suggested some genetic components of neovascular AMD and PCV are different. Therefore, studies involving both neovascular AMD and PCV will help to decipher the genetic similarities and differences between these two clinical phenotypes. Moreover, epigenetics may also be involved in the development of AMD and PCV [27, 28]. Therefore, detailed assessment of the environmental and constitutional factors followed by interaction analysis with genetic factors would help to better assess the risk of developing AMD and PCV.

Association of the *CFH* gene with AMD has revealed the involvement of genes in the complement pathway in AMD pathogenesis. Subsequently, candidate gene association analyses suggested that genes encoding other complement components were also associated with AMD and/or PCV, including *complement component 2* (*C2*), *complement factor B* (*CFB*), *complement component 3* (*C3*), and *complement factor I* (*CFI*) [29–32]. The complement system is part of the innate immune system in human and plays a role in clearing pathogens from organisms and eliminating immune complex. Essentially three pathways activate the complement system: the classical, alternative and lectin pathways. Regardless of their initiation points, these three pathways merge at the activation of C3 by a C3 convertase, which cleaves C3 into C3a and C3b. Binding of C3b and a C3 convertase forms a C5 convertase, cleaving C5 into C5a and C5b. The C5 protein plays a role in the pathogenesis of AMD as evident by its presence in drusen [33, 34] and the elevation of C5a in peripheral blood of AMD patients [35, 36]. Secretion of the angiogenic factor vascular endothelial growth factor from retinal pigment epithelium cells was up-regulated by C5a both in vitro and in vivo [37, 38]. C3a and C5a in the RPE and choroid was upregulated in laser-induced CNV mice models, whilst genetic ablation of C3a and C5a receptors caused reduction of laser-induced CNV in mice models [37]. All these evidences suggested that C5a may be involved in the development of CNV, which is the hallmark of neovascular AMD.

So far, the role of *C5* as an AMD-associated gene remains uncertain. No significant association between *C5* and advanced AMD was identified in Caucasians in the studies of Yates et al. and Maller et al. [30, 31], while a significant association was identified in another Caucasian cohort [39]. In contrast, the association between *C5* and PCV has not been reported in the literature. We have previously shown in ethnic Chinese the genetic susceptibility of neovascular AMD and PCV with genes in the complement pathways, *CFH*, *SERPING1*, *C2*, and *C3* [23, 40–42]. Herein we performed a haplotype-tagging SNP-based association analysis to evaluate the association of *C5* with neovascular AMD and PCV in Chinese.

## Methods

### Study participants

The study protocol was approved by the Ethics Committee on Human Research, The Chinese University of Hong Kong. The study procedures followed the tenets of the Declaration of Helsinki. All study subjects provided written informed consent.

This study involved a total of 708 unrelated Chinese study subjects consisting of 200 neovascular AMD patients, 233 PCV patients and 275 healthy controls. They were recruited from the Hong Kong Eye Hospital and the Eye Centre of the Prince of Wales Hospital, Hong Kong. The study subjects had been involved and described in our previous reports [19, 20, 40–44]. In brief, all patients underwent complete ophthalmic examinations, including visual acuity, ocular tonometry, slit-lamp biomicroscopy, ophthalmoscopy, fundus photography, fluorescein angiography, and indocyanine green angiography (ICGA). All AMD patients had been diagnosed as having neovascular AMD in at least one eye. PCV was diagnosed by characteristic polypoidal lesions from the choroid on ICGA. Patients with presence of both CNV and PCV lesions in the same or fellow eye were excluded. Unrelated control subjects were recruited from people who attended the clinic for eye examinations and aged older than 60 years with no signs of AMD, PCV or other eye diseases, except mild senile cataracts and mild refractive errors. Demographic information has been summarized in Table 1.

**Table 1:** Demographic features of the Study Subjects

	AMD (*n* = 200)	PCV (*n* = 233)	Control (*n* = 275)	Comparison (P value)	
				AMD-Control	PCV-Control
Male (%)	110 (55.0)	162 (69.5)	121 (44.0)	0.02	< 0.001
Age (years)					
Mean ± SD	75.3 ± 7.7	68.5 ± 9.0	74.3 ± 7.6	0.16	< 0.001
Age range	50–94	53–90	60–94		

*AMD*= age-related macular degeneration, *PCV*= polypoidal choroidal vasculopathy, *SD*= standard deviation

### SNP selection and genotyping

Six SNPs (rs2269066, rs17611, rs1548782, rs10985126, rs12237774 and rs1017119) were selected to cover and tag the entire *C5* gene. SNP data for Han Chinese in Beijing population (CHB) was obtained from the International HapMap Project (http://hapmap.ncbi.nlm.nih.gov/, HapMap Genome Browser release #27). The tagging SNPs were selected by a pairwise method with a minor allele frequency cutoff of 0.1 and r^2^ cutoff of 0.8, adopting a functional ranking system wherein non-synonymous SNPs were selected preferentially, followed by synonymous SNPs, SNPs in 5′ untranslated regions, SNPs in 3′ untranslated regions, and SNPs in introns.

Genomic DNA was extracted from peripheral blood using a DNA extraction kit (Qiagen QIAamp DNA Blood Mini kit, Qiagen, Hilden, Germany) according to the manufacturer’s protocol. The six tagging SNPs were genotyped using *TaqMan* genotyping assays (Applied Biosystems [ABI], Foster City, CA) on a Roche LightCycler® 480 Real-Time PCR System (Roche, Switzerland) according to the manufacturer’s instructions.

### Statistical analysis

Age and gender difference between cases and controls were assessed using the independent *t-*test and the chi-square test, respectively, with SPSS software version 20.0 (SPSS Inc., Chicago, IL). Hardy-Weinberg Equilibrium (HWE) of individual SNPs were tested using PLINK (v1.07, http://zzz.bwh.harvard.edu/plink). Allelic and genotypic association of all SNPs with neovascular AMD and PCV were evaluated by the chi-square test or Fisher’s exact test, and age and gender were adjusted by logistic regression in PLINK. The wild type allele was taken as reference for estimating odds ratio (OR) and 95% confidence interval (CI). Haplotype association analysis was performed using the confidence interval method in Haploview (v4.2, http://www.broad.mit.edu/mpg/haploview).

As the proteins encoded by *C5* and *C3* interact biologically in the complement pathway, pairwise interaction analysis between the tagging SNPs of the two genes was conducted using the epistasis option in PLINK to assess potential gene-gene interaction. Genotype data of the *C3* tag SNPs were obtained from our previous study [42]. Also, to evaluate the gene-gene interaction between *C5* and other candidate genes of AMD and PCV, genotypic data of the *SERPING1* [40], *C2*-*CFB*-*RDBP*-*SKIV2L* [41], *CETP* [43], *ABCG1* [44], *PGF* [19], *ANGPT2* [20], *CFH* [43] and the *HTRA1* [43] genes were extracted from our previous studies for interaction analyses. Moreover, in view of the detection of gene-gender interaction between *C3* and gender [42], we also performed SNP-gender interaction analysis for the *C5* gene using logistic regression. *P* value of less than 0.05 was considered statistically significant.

## Results

The age and gender distribution were significantly different between patients and controls (Table 1). They were thus adjusted in the association analyses using logistic regression.

### Individual SNP association analysis

In the International HapMap Project for CHB population, the 6 selected SNPs captured all alleles in the *C5* gene with a minor allele frequency larger than 0.1 and a mean r^2^ of 0.94. All SNPs were successfully genotyped and conformed to the HWE (*P* > 0.05) in both cases and controls. No significant difference of the allelic frequencies for these SNPs was observed in neovascular AMD and PCV compared with controls (P > 0.05, Table 2). None of the SNPs showed a significant association with neovascular AMD or PCV after adjusting for age and gender (all *P* values > 0.05). Also, no significant association was identified with neovascular AMD and PCV under dominant and recessive models (all P values > 0.05). Furthermore, none of the SNPs showed significant differences between neovascular AMD and PCV (Table 2).

**Table 2:** Allelic association of SNPs in *C5* with neovascular AMD and PCV

SNP	Location	Codon change	Minor allele	Minor allele frequency			Allelic association					
				AMD	PCV	Control	AMD-control		PCV-control		AMD-PCV	
				(*n* = 400)	(*n* = 466)	(*n* = 550)	P	OR (95% CI)	P	OR (95% CI)	P	OR (95% CI)
rs1017119	intron 2	–	C	0.16	0.15	0.14	0.54	1.12 (0.78–1.61)	0.70	1.07 (0.76–1.52)	0.82	0.96 (0.66–1.39)
rs10985126	exon 11	G385G	C	0.24	0.23	0.24	0.83	0.97 (0.72–1.31)	0.66	0.94 (0.70–1.25)	0.84	0.97 (0.71–1.33)
rs1548782	intron 18	–	T	0.21	0.24	0.20	0.83	1.04 (0.75–1.42)	0.15	1.25 (0.93–1.68)	0.26	1.20 (0.87–1.66)
rs17611	exon 19	V802I	G	0.41	0.42	0.41	0.85	0.98 (0.75–1.27)	0.67	1.06 (0.82–1.36)	0.57	1.08 (0.82–1.42)
rs2269066	intron 30	–	T	0.20	0.19	0.22	0.44	0.88 (0.64–1.21)	0.28	0.84 (0.62–1.15)	0.79	0.96 (0.68–1.34)
rs12237774	exon 34	A1422A	T	0.18	0.17	0.20	0.54	0.90 (0.65–1.26)	0.18	0.80 (0.58–1.11)	0.50	0.89 (0.62–1.26)

*AMD*= age-related macular degeneration, *CI*= confidence interval, *OR*= odds ratio, *PCV*= polypoidal choroidal vasculopathy, *SNP*= single nucleotide polymorphism

### Linkage disequilibrium (LD) and haplotype analysis

**Fig. 1:**
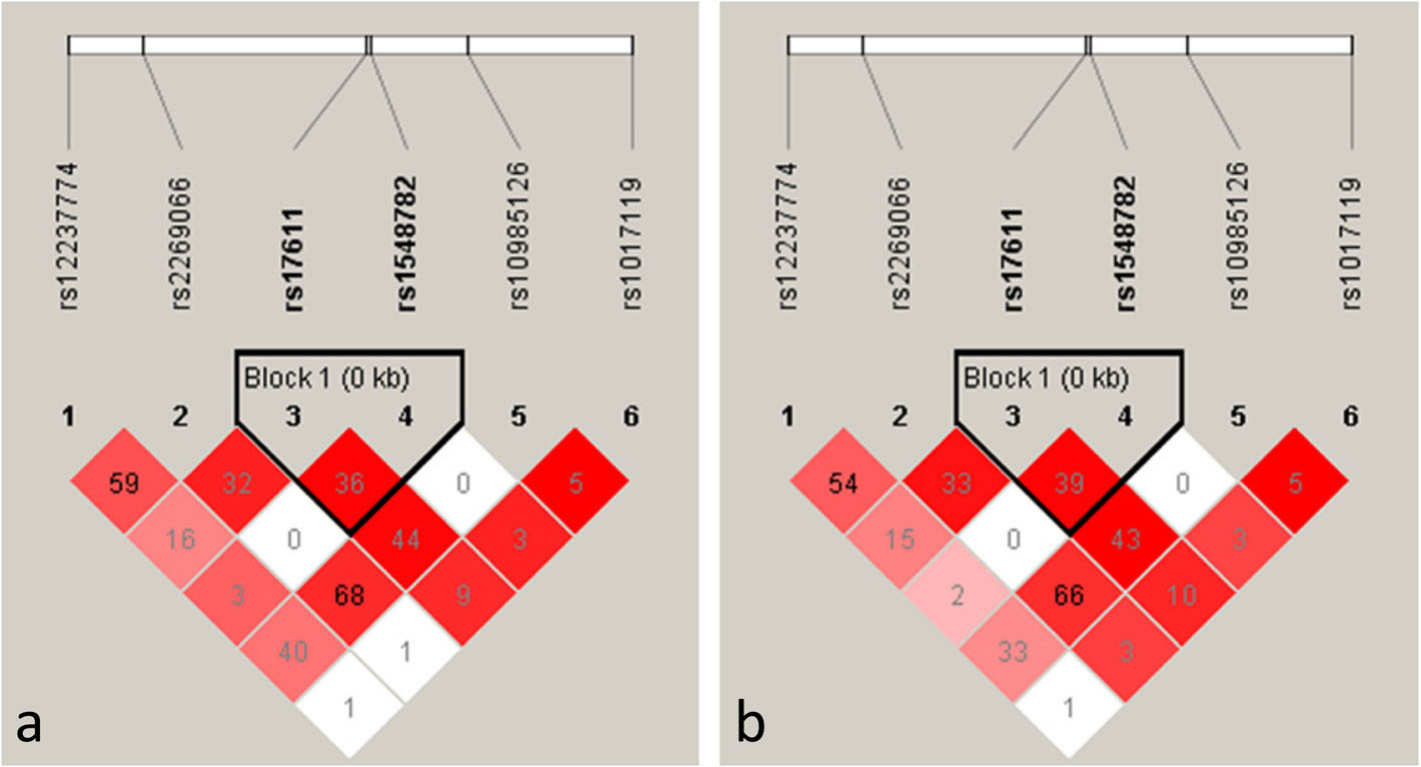
Linkage disequilibrium (LD) structure of *C5* for neovascular AMD (**a**) and PCV (**b**). LD was measured using data from all controls and neovascular AMD or PCV in the present study. The confidence interval method was used to define the haplotype blocks. The LD (r^2^) between any two SNPs is listed in the cross cells. AMD: age related macular degeneration, PCV: polypoidal choroidal vasculopathy, SNPs: single nucleotide polymorphisms

LD analysis across *C5* using these 6 SNPs showed that 2 SNPs, rs17611 and rs1548782 were included in one haplotype block in both neovascular AMD and PCV (Fig. 1). Three haplotypes defined by these two SNPs were identified. None of the haplotypes was significantly associated with neovascular AMD or PCV (*P* > 0.05, Table 3), and their distributions between the two disease groups were similar.

**Table 3:** Haplotype associations of *C5* with neovascular AMD and PCV

Haplotype	Frequency				Association (P)		
rs17611-rs1548782	AMD	PCV	Control		AMD-Control	PCV-Control	AMD-PCV
1 A-A	237.9 (0.59)	268.1 (0.58)	322.8 (0.59)		0.81	0.71	0.85
2 G-T	83.9 (0.21)	112.9 (0.24)	110.8 (0.20)		0.76	0.12	0.17
3 G-A	78.1 (0.20)	84.9 (0.18)	115.2 (0.21)		0.59	0.28	0.36

*AMD*= age related macular degeneration, *PCV*= polypoidal choroidal vasculopathy

### Interaction analysis between SNPs in *C5* and other genes, and between *C5* and gender

Genotypic data of major SNPs in the C3 (rs17030) [42], *SERPING1* (rs1005510 and rs11603020) [40], *C2*-*CFB*-*RDBP*-*SKIV2L* (rs547154, rs760070, rs429608 and rs453821) [41], *CETP* (rs3764261) [43], *ABCG1* (rs57137919 and rs225396) [44], *PGF* (rs2268615 and rs2268614) [19], *ANGPT2* (rs13255574, rs4455855, rs13269021, and rs11775442) [20], *CFH* (rs800292) and *HTRA1* (rs11200638) [43] genes were extracted from our previous studies for interaction analysis with each of the 6 selected *C5* SNPs in this study. However, pairwise epistasis analysis revealed no significant SNP-SNP interaction between *C5* and *C3* or any other genes for neovascular AMD and PCV (all *P* values for the interaction term were > 0.05). Also, there was no significant SNP-gender interaction for *C5* (*P* > 0.1).

## Discussion

In this study, we evaluated the associations of 6 haplotype-tagging SNPs in the *C5* gene with neovascular AMD and, for the first time, in PCV in a Chinese cohort. Although there are evident involvement of the C5 protein in neovascular AMD as the fragment C5a increased the risk of CNV [37, 38], none of the *C5* tagging SNPs or haplotypes showed significant association with neovascular AMD or PCV (*P* > 0.05). In addition, we found no significant SNP-SNP interaction between *C5* and *C3* or other involved genes in neovascular AMD or PCV.

Our results are consistent with those in previous studies on AMD patients of non-Chinese ethnicity. Yates et al. reported no association between *C5* SNPs and advanced AMD, including geography atrophy and neovascular AMD, in a Caucasian population of 603 cases and 350 controls [30]. A subsequent study showed that the tagging SNPs across *C5* were not associated with advanced AMD in an European population of 1238 cases and 934 controls [31]. Later, Baas et al. performed a comprehensive analysis between *C5* SNPs and several forms of AMD, including early AMD, geography atrophy and neovascular AMD, in four independent studies [39]. Although significant association between *C5* and AMD was identified in the original study on a Dutch population (AMRO-NL study), this association could not be replicated in the other three study cohorts from the Netherlands, the United Kingdom, and the United States [39]. The incidence of neovascular AMD was higher in the AMRO-NL cohort (50.4%), as compared with that of the other three cohorts (5.8, 43.1 and 42.8%, respectively) [39], which might have contributed to the different results. Here we made the first attempt to investigate *C5* in PCV. We did not find significant associations between individual *C5* SNPs and PCV or neovascular AMD. Our results thus rule out a definite role of *C5* in neovascular AMD and PCV, although further confirmation in larger study cohorts should be warranted.

AMD and PCV are multifactorial late-onset diseases with genetic susceptibility, environmental factors being the major risk factors. Gene-gene interactions of *CFH* and *ARMS2*, and interaction between genes and environmental risk factors, such as smoking and gender, have been found to be implicated in the disease risk of AMD and PCV [42, 45–48]. There was also evidence to suggest the existence of epistasis in AMD [49]. Although the exact mechanism for the epistasis was unclear, a combination of two SNPs, rs1394608 in *SGCD* and rs3743175 in *SCAPER*, was identified to be associated with AMD in the analysis of a genome-wide case-control data set [49]. Since C5 and C3 interact with each other biologically in the complement system, we evaluated the role of epistasis between *C5* SNPs and *C3* SNPs. However, we identified no significant SNP-SNP interaction for neovascular AMD and PCV between the tagging SNPs in *C5* and *C3*. Also, no gene-gene interaction was identified between *C5* and *C2*-*CFB*-*RDBP*-*SKIV2L*, *SERPING1*, *CETP*, *ABCG1*, *PGF*, *ANGPT2*, *CFH* or *HTRA1*. Moreover, no gene-gender interaction was identified for *C5* in AMD or PCV.

This study provides an evaluation of the *C5* gene and interaction between *C5* and *C3* and other candidate genes in neovascular AMD and PCV in a Chinese cohort. However, several limitations should be taken into account when interpreting the negative findings. First, the sample size in each group was relatively small. Our samples provided a statistical power of approximately 50% to rule out the null hypothesis of no association at the alpha level of 0.05, assuming a modest odds ratio of 1.5. Therefore, larger study cohorts are needed to confirm the lack of association between *C5* and neovascular AMD or PCV in Chinese. Second, the mean age and gender ratios were significantly different between the case and control groups, especially between PCV and controls. Therefore, further work should include more age-matched female patients and male controls so that there would be age and gender balance in both patients and controls. Third, the smoking status and clinical parameters of some study subjects were not available, therefore these factors could not be incorporated in the data analysis of this study. New recruitment work is ongoing to resolve these issues.

## Conclusions

This study suggests that the *C5* SNPs did not have a significant association with the disease risk of neovascular AMD and PCV in the Hong Kong Chinese cohort. In addition, no significant epistasis was identified between *C5* and gender or SNPs in other genes, including *C2*-*CFB*-*RDBP*-*SKIV2L*, *C3*, *SERPING1*, *CETP*, *ABCG1*, *PGF*, *ANGPT2*, *CFH* or *HTRA1*. In view of the limited sample size in this study, further studies in large samples from different populations are warranted to confirm the role of the *C5* gene in the genetic susceptibility of neovascular AMD and PCV.

## Data Availability

Datasets related to this study will be available from the corresponding author upon request.
